# A CMOS RF Receiver with Improved Resilience to OFDM-Induced Second-Order Intermodulation Distortion for MedRadio Biomedical Devices and Sensors

**DOI:** 10.3390/s21165303

**Published:** 2021-08-05

**Authors:** Yongho Lee, Shinil Chang, Jungah Kim, Hyunchol Shin

**Affiliations:** Department of Electronics Convergence Engineering, Kwangwoon University, Seoul 01897, Korea; dldyd91@kw.ac.kr (Y.L.); starv79@kw.ac.kr (S.C.); ccog4@kw.ac.kr (J.K.)

**Keywords:** RF receiver, blocker, second-order intermodulation (IM2), orthogonal frequency division modulation (OFDM), CMOS, MedRadio, medical implanted communication service (MICS), biomedical device, biosensors

## Abstract

A MedRadio RF receiver integrated circuit for implanted and wearable biomedical devices must be resilient to the out-of-band (OOB) orthogonal frequency division modulation (OFDM) blocker. As the OFDM is widely adopted for various broadcasting and communication systems in the ultra-high frequency (UHF) band, the selectivity performance of the MedRadio RF receiver can severely deteriorate by the second-order intermodulation (IM2) distortion induced by the OOB OFDM blocker. An analytical investigation shows how the OFDM-induced IM2 distortion power can be translated to an equivalent two-tone-induced IM2 distortion power. It makes the OFDM-induced IM2 analysis and characterization process for a MedRadio RF receiver much simpler and more straightforward. A MedRadio RF receiver integrated circuit with a significantly improved resilience to the OOB IM2 distortion is designed in 65 nm complementary metal-oxide-semiconductor (CMOS). The designed RF receiver is based on low-IF architecture, comprising a low-noise amplifier, single-to-differential transconductance stage, quadrature passive mixer, trans-impedance amplifier (TIA), image-rejecting complex bandpass filter, and fractional phase-locked loop synthesizer. We describe design techniques for the IM2 calibration through the gate bias tuning at the mixer, and the dc offset calibration that overcomes the conflict with the preceding IM2 calibration through the body bias tuning at the TIA. Measured results show that the OOB carrier-to-interference ratio (CIR) performance is significantly improved by 4–11 dB through the proposed IM2 calibration. The measured maximum tolerable CIR is found to be between −40.2 and −71.2 dBc for the two-tone blocker condition and between −70 and −77 dBc for the single-tone blocker condition. The analytical and experimental results of this work will be essential to improve the selectivity performance of a MedRadio RF receiver against the OOB OFDM-blocker-induced IM2 distortion and, thus, improve the robustness of the biomedical devices in harsh wireless environments in the MedRadio and UHF bands.

## 1. Introduction

An RF transceiver integrated circuit operating in the MedRadio band is widely employed for biomedical devices and sensors, as the wireless communication is essentially needed between the implanted or body-worn medical devices and outside controllers. The wireless connectivity for the biomedical devices is used to exchange the diagnostic and therapeutic data. Its non-invasiveness significantly improves the patient’s comfortability by avoiding unnecessary painful surgical operations. The MedRadio band was assigned in 2009 [1], in the frequency band of 401–406 MHz providing a total 5 MHz of contiguous spectrum on a secondary and non-interference basis. The MedRadio rule was amended later in 2011 to allow networking of the devices and controllers and referred to as a medical micropower network (MMN) [2]. Since then, numerous implanted and wearable medical devices equipped with the MedRadio RF transceiver have been reported in literature, such as a wireless bio-signal monitoring system [3], an implanted cardiac defibrillator [4], an implanted pacemaker [5], an intraocular pressure monitoring system [6], physiological state monitoring prosthetic teeth [7,8], wireless position sensing system in total hip replacement surgery [9], capsule endoscopy [10,11], and so on.

The RF communication channel of the MedRadio is accessed on a shared or secondary basis. Hence, they must be robust to interferers coming from nearby other authorized primary users [12]. For mitigating the interferences, system-level techniques, such as error detection and correction via proper channel-coding, listen-before-talk (LBT), and re-transmission via a frequency monitoring and classification process, are widely employed [1]. Yet, even though the system-level interference mitigation techniques are employed, additional circuit-level techniques are still needed in the RF transceiver design to improve the overall interference resilience. Unfortunately, however, most previous MedRadio RF integrated circuits, either transceivers [4,5,12,13] or receivers [14,15,16,17], did not address those issues nor present proper circuit designs for its mitigation. Ba et al. [12] and Cha et al. [14] indeed mentioned the in-band interference issue and described the adjacent channel rejection (ACR) and the third-order intercept power (IIP3) performances. However, their studies were still limited only to the in-band interference situation and cannot be generalized to the out-of-band interference situation. It is also interesting to note that Cho et al. [18] addressed the very-high frequency (VHF) band interference issue in their dual-band RF receiver and demonstrated a blocker tolerance level of −45 dBm for the MedRadio receiver against the VHF body-channel communication interference. However, their result was also limited to the VHF-band single-tone interference issue and not applicable to the UHF-band multi-tone interference issue. It is also worth noting that non-conventional signal modulation techniques can be also effective for the interference resilience improvement. Novel approaches such as the two-tone modulation [19] and the spread-spectrum modulation [20] were proven effective in 900 MHz transceivers. Yet, they were only applicable to the on-off keying (OOK) modulation and not to the constant-envelope modulation that is more widely adopted by MedRadio devices.

The modern UHF and VHF bands are crowded with a variety of multi-tone signals. They always can be seen as unwanted strong interferers to the MedRadio biomedical devices and sensors. For example, the fifth-generation new radio (5G NR) band encompasses 600–800 MHz UHF band in its frequency range 1 (FR1) band. Additionally, digital broadcasting standards such as the advanced television systems committee (ATSC) of the United States and Korea [21], the digital video broadcasting (DVB) of Europe [22], and the intelligent service digital broadcasting (ISDB) of Japan [23] are serviced in the VHF band of 54–216 MHz and UHF band of 470–860 MHz. Moreover, these interferences are likely to adopt the orthogonal frequency division modulation (OFDM) signaling for high data rate and strong multi-path fading resilience. When an equally spaced multi-tone OFDM signal comes into the MedRadio device as an interferer, numerous intermodulation distortion components will be created by the multiple subcarrier tones, even though they are out of the band. Thus, in modern MedRadio receiver design, it is essential to analyze the effects of the out-of-band (OOB) interference on the receiver’s signal-to-noise ratio (SNR) and to design circuits for mitigating its effects. Note that this issue usually cannot be neglected in typical MedRadio receivers even though the interference exists out of the band. We cannot expect sufficient filtering and attenuation for the OOB interference at the RF front end because typical MedRadio antennas show wideband characteristics [11], a complex of high-order matching and filtering at the RF front end may not be favored for low cost and small form factor realization [10], and the LNA in a receiver integrated circuit is not typically band-specific because resistive loads are frequently adopted rather than bulky inductors for the small silicon area.

In this work, we analyze the intermodulation distortion effects induced by the OOB OFDM interferer on the MedRadio receiver and, then, present a design of a CMOS RF receiver by focusing on the second-order intermodulation (IM2) distortion tolerance improvement. This work is carried out based on our prior work [17], which is expanded by adding a complex bandpass filter to realize a low-IF receiver and an IM2 calibration circuit to minimize the OOB OFDM-induced IM2 distortions.

## 2. Analysis of OFDM-Induced IM2 Distortion Effects

**Figure 1:**
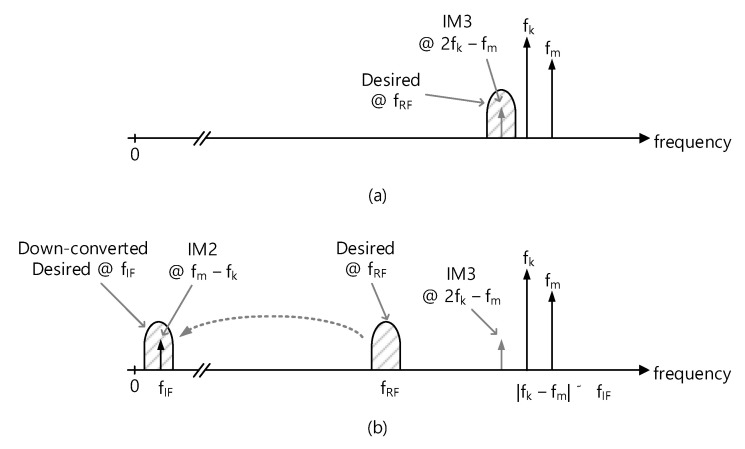
Two-tone blocker effects. (**a**) Third-order intermodulation effect for the close-in blocker, (**b**) second-order intermodulation effects for the far-out blocker.

Let us assume that a desired MedRadio RF signal comes into the receiver along with an OOB OFDM blocker. Since the OFDM signal comprises multiple subcarrier tones that are independently modulated and equally spaced in the frequency domain, any combination of two subcarrier tones within the entire OFDM signal will create multiple intermodulation distortion components. Figure 1a shows the first situation such that two subcarrier tones at *f_k_* and *f_m_* create the third-order intermodulation (IM3) distortion at 2*f_k_* − *f_m_*. The resulting IM3 falls inside the desired RF channel at *f_RF_*, directly leading to SNR degradation at *f_RF_*. Considering that the typical subcarrier spacing is in the range of a few 100 Hz to a few kHz for the communication and broadcasting signals, this effect is only possible when the two subcarrier tones are very close to *f_RF_*. Such a close blocker signal will be likely to exist inside the desired band. Then, the in-band interference mitigation techniques such as the channel-scan-and-search-based LBT will be effective enough for its mitigation.

On the other hand, when the OFDM blocker signal is far away from *f_RF_* as shown in Figure 1b and is, thus, likely to be out of the MedRadio band, then its IM3 tone cannot directly affect the desired band at *f_RF_*. Yet, the IM2 distortion at *f_m_* − *f_k_* will fall in the down-converted IF or baseband channel at *f_IF_* and lead to the SNR degradation. The LBT technique that works for the close blocker signal will not work for this situation because the blocker signal is located far away out of the band and is, thus, likely out of the channel scan range.

**Figure 2:**
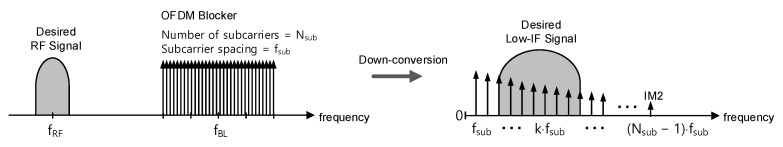
IM2 distortions induced by the multi-tone OFDM blocker.

It should be noted that a variety of communication and broadcasting services that are being offered worldwide in the UHF and VHF bands employs the OFDM signaling. Hence, the IM2 distortions induced by the OOB OFDM blocker signal need to be analyzed rigorously to accurately assess the SNR degradation in the MedRadio receiver. Figure 2 illustrates that the OOB OFDM blocker signal at *f_BL_* appears beside the desired MedRadio RF signal at *f_RF_*. Let the number of subcarriers and subcarrier spacing of the OFDM blocker are *N_sub_* and *f_sub_*, respectively. For example, in the ATSC 3.0 standard [21], given the channel bandwidth of 6 MHz, *f_sub_* is 843.7, 421.8, and 210.9 Hz depending on the OFDM FFT modes of 8K (*N_sub_* = 6913), 16K (*N_sub_* = 13,826), and 32K (*N_sub_* = 27,649), respectively. Similarly, we can find that *f_sub_* is 279–8929 Hz for DVB-T2 [22], and 992–3968 Hz for ISDB-T [23].

As shown in Figure 2, any combination of two subcarrier tones out of the total *N_sub_* subcarrier tones will create (*N_sub_* − 1) of IM2 tones at the baseband, starting from the first at *f_sub_* to the last at (*N_sub_* − 1)·*f_sub_*. If the desired IF signal band overlaps with the IM2 tones, the total IM2 distortion power can be computed by integrating its power spectral density (PSD) over the IF channel bandwidth. Then, the SNR degradation caused by the OFDM-induced IM2 distortions can be accurately evaluated. For example, the IF signal bandwidth of Figure 2 encompasses the 3rd IM2 tone at 3⋅*f_sub_* through the 10th IM2 tone at 10⋅*f_sub_*. Thus, the total IM2 distortion power should be computed by integrating from the 3rd through the 10th IM2 distortion power.

The well-known input-referred second-order intercept point power (*IIP_2_*) of an RF receiver is expressed as

(1) $$IIP_{2}=P_{in}+\left(P_{out}-P_{OIM2}\right)$$

where *P_in_* is the input power, *P_out_* is the output power, and *P_OIM2_* is the output-referred IM2 distortion power, all expressed in dB or dBm. When a OFDM blocker signal that has the total power of *P_in.total_* is fed to a receiver having *IIP_2_* and a power gain of *G_P_*, the input-referred IM2 distortion power *P_IIM2_* (= *P_OIM2_ − G_P_*) can be expressed by

(2) $$P_{IIM2}=2P_{in.total}-IIP_{2}$$

If the total number of subcarriers is *N_sub_*, the power of each subcarrier *P_in.sub_* is given by

(3) $$P_{in.sub}=P_{in.total}-10log(N_{sub})$$

Now, let us compute the total input-referred IM2 distortion power by integrating the IM2 PSD over the band of interest. Ranjan et al. in their OFDM distortion analysis [24] derived an analytic expression of the IM2 PSD by assuming that IM2 tones at the baseband are uncorrelated with the original causative OFDM data. Although the exact PSD expression (Equation (12) of [24]) is not repeated here, we can find that the numerical integration of PSD can be simply carried out by collecting and adding the subcarrier PSD components at their center frequencies without a significant loss in generality and accuracy. Let us take an example of the 3rd *P_IIM2_* illustrated in Figure 2, which is the first IM2 tone that exists in the IF band. We can find that the 3rd *P_IIM2_* is created by (*N_sub_* − 3) of two subcarrier tones that are apart by 3·*f_sub_* in the original OFDM blocker signal. This observation can be generalized such that the *k*-th IM2 power *P_IIM2.kth_* can be obtained by *k*-th *P_IIM2_* multiplied by the total number of the subcarrier pairs responsible for creating the *k*-th IM2 tone. It is expressed by

(4) $$10^{\frac{P_{IIM2.kth}}{10}}=\left(N_{sub}-k\right)\cdot 10^{\frac{2P_{in.sub}-IIP2}{10}}$$

If the band of interest covers from the *k_i_*-th IM2 tone through the *k_f_*-th IM2 tone, then, the total input-referred IM2 power *P_IIM2.total_* that exists within the band of interest can be calculated by summing (*N_sub_ − k*) of *P_IIM2.kth_* of (4) from the first *k_i_*-th through the last *k_f_*-th. It is expressed as follows

(5) $$10^{\frac{P_{IIM2.total}}{10}}=\sum_{k=k_{i}}^{k_{f}}\left(\left(N_{sub}-k\right)\cdot 10^{\frac{2P_{in.sub}-IIP2}{10}}\right)$$

Equation (5) can be written again in dB as follows

(6) $$P_{IIM2.total}=\left(2P_{in.sub}-IIP_{2}\right)+10\log\left(\sum_{k=k_{i}}^{k_{f}}\left(N_{sub}-k\right)\right)$$

By substituting *P_in.sub_* of (6) with (3), Equation (6) can be arranged as

(7) $$P_{IIM2.total}=2\left(P_{in.total}-P_{offset}\right)-IIP_{2}$$

where *P_offset_* is given by

(8) $$P_{offset}=10log\frac{N_{sub}}{\sqrt{\sum_{k=k_{i}}^{k_{f}}\left(N_{sub}-k\right)}}$$

Equations (7) and (8) imply that the IM2 distortion power induced by the OFDM signal can be equivalently evaluated by the IM2 distortion power induced by the two-tone blocker signal as long as the two-tone blocker signal power is set to be lower than the original OFDM blocker signal power *P_in.total_* by *P_offset_*.

This finding greatly simplifies the simulation and characterization of the OFDM-induced IM2 distortion in an RF receiver. Involvement of a multi-carrier OFDM signal in RF circuit simulations will typically require a sophisticated OFDM signal modeling, a very complex numerical analysis method, and a long simulation time. Thus, it is very time-consuming and impractical to examine the OFDM-induced IM2 effects in RF circuit design. However, if the OFDM blocker signal can be replaced simply by an equivalent two-tone blocker signal by considering an offset parameter *P_offset_*, the whole simulation process will become much simpler and more convenient because we can use the conventional time- and frequency-domain circuit simulation methods. Thus, these analytic results enable us to evaluate the OFDM-induced IM2 effects very efficiently when only examining an equivalent two-tone blocker induced IM2 effects.

Let us take an example of 16K FFT mode of ATSC 3.0 [21], which has *N_sub_* = 13,825 and *f_sub_* = 421.875 Hz. When the IF band of a low-IF MedRadio receiver resides in 150–450 kHz, the IM2 tones appearing within the band are from *k_i_* = 356th to *k_f_* = 1066th components. Then, *P_offset_* is computed to +6.56 dB by (8). It implies that if an ATSC blocker of −10 dBm is injected to a receiver and induces IM2 distortion at the baseband, the same amount of IM2 distortion power is also induced by a simple two-tone blocker of −16.56 dBm. Moreover, assuming the input signal power of the receiver is −50 dBm, and the receiver *IIP_2_* is +30 dBm, *P_IIM2.total_* will be −63.1 dBm (=2 × (−10 − 6.56) − 30) according to Equation (7), and the resulting SNR will be +13.1 dB as the input signal power is −50 dBm and the input-referred distortion power *P_IIM2.total_* is −63.1 dBm

## 3. Designs

**Figure 3:**
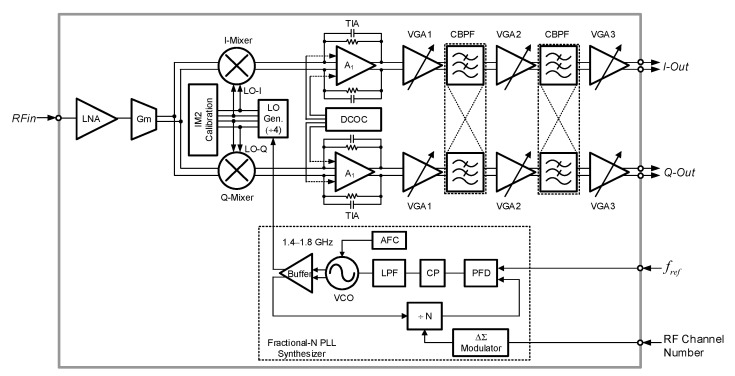
The MedRadio RF receiver architecture.

Figure 3 shows the architecture of the MedRadio RF receiver. It is designed in an RF CMOS process. The receiver comprises a low-noise amplifier (LNA), transconductance (Gm) stage, quadrature down-conversion mixer with IM2 calibration, trans-impedance amplifier (TIA) with dc offset calibration (DCOC), three-stage variable gain amplifiers (VGAs), complex bandpass filter (BPF), fractional-N phase-locked loop (PLL) synthesizer, and a divide-by-4 local oscillator (LO) generator.

The receiver takes a low intermediate-frequency (IF) architecture employing a quadrature single down-conversion scheme. The IF frequency is set equal to the channel bandwidth of 300 kHz so that the down-converted IF band resides in 150–450 kHz.

**Figure 4:**
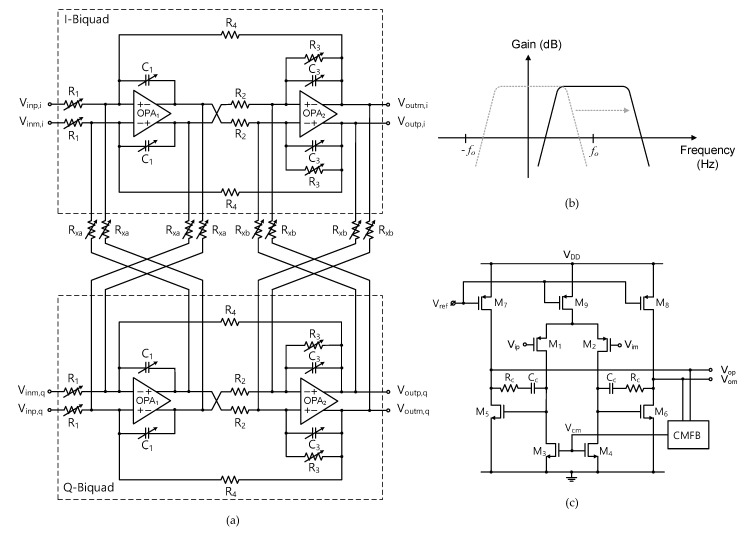
Complex biquad. (**a**) Block diagram, (**b**) transfer characteristics, (**c**) operational amplifier schematic.

The image band of the desired RF signal is rejected by the complex BPF. As shown in Figure 3, the complex BPF comprises two-stage complex biquads and three-stage VGAs. The detailed block diagram of the single-stage complex biquad is shown in Figure 4a. The unit biquad is based on a modified Tow-Thomas low-pass filter (LPF) structure in which the lossless integrator block (OPA_1_, C_1_) is put before the lossy integrator block (OPA_2_, R_3_, C_3_). Since the second-order filtering is given by the unit biquad, the overall two-stage complex BPF presents a fourth-order bandpass filtering characteristics. Figure 4b illustrates how the low-pass characteristics of the unit biquad are translated to the complex bandpass characteristics. The cross-interconnecting resistors R_xa_ and R_xb_ that are placed between the I- and Q-path biquads as shown in Figure 4a shift the complex conjugate poles to real poles, resulting in the original low-pass filter response being shifted to the desired complex bandpass filter response, which gives the image rejection capability [25]. For optimal image rejection performance, the center frequency *f_o_* is equally set to the channel bandwidth. As a result, the image component at the negative frequency band at −*f_o_* is suppressed with respect to the wanted positive frequency band at +*f_o_*. To cope with process variability and also to support variable channel bandwidth modes, key performance parameters of the complex biquad are tunable by realizing the on-chip resistors and capacitors in a switched value structure. The resulting switched tuning ranges are given by 34–136 kΩ with 2-bit control for R_1_, 82–103 kΩ with 3-bit control for R_3_, and 0.6–7.35 pF with 4-bit control for C_1_ and C_3_, while R_2_ and R_4_ are fixed to 135 kΩ. Then, the overall complex BPF shows a tunable gain of −22–+45 dB, tunable center frequency of 0.25–3 MHz, tunable bandwidth of 0.23–2.7 MHz, and tunable quality factor of 0.9–1.1. In addition, the passband flatness is also tunable by independently controlling the on-chip R_xa_ and R_xb_ in the range of 78–106 kΩ so that the band-edge gain difference is adjusted in the range of −1–+1 dB [25].

Figure 4c is the schematic of the operational amplifiers OPA_1_ and OPA_2_. It is a fully differential two-stage amplifier with a dc common-mode feedback (CMFB). The first stage is a differential pair having *p*-FET input pair of M_1,2_ and *n*-FET active load of M_3,4_, and the second stage is *n*-FET common-source stage of M_5,6_. Designed parameters of the FET’s gate width/length are 200/0.2 μm for M_1,2_, 80/0.5 μm for M_3,4_, 64/0.2 μm for M_5,6_, 192/0.5 μm for M_7,8_, and 480/0.5 μm for M_9_. The dc bias currents are 42 μA for M_9_ and 18 μA for M_7,8_. The frequency compensating R_C_ and C_C_ are 4 kΩ and 0.9 pF, respectively.

The LO signal is generated by the fractional-N PLL frequency synthesizer. A 20-bit digital delta-sigma (Δ∑) modulator is employed for the fractional-N frequency generation. Details of this PLL can be found in the author’s prior work [26]. The voltage controlled oscillator (VCO) covers 1.4–1.8 GHz, which is 4× higher than the MedRadio RF band of 401–406 MHz. The automatic frequency calibration (AFC) searches for an optimal sub-band out of the 32 sub-bands through 5-bit switched capacitor bank of the VCO. The divide-by-4 LO generator circuit after the VCO buffer generates I/Q LO signals with 25% duty cycle for driving the mixer FETs. The 25% duty-cycle LO signal improves the conversion gain and noise figure performances of the quadrature mixer [17].

**Figure 5:**
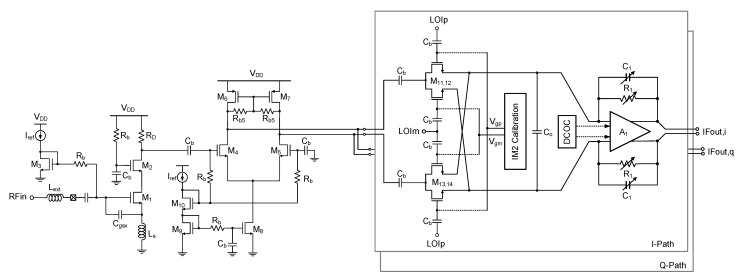
Circuit schematic of the RF front end.

Figure 5 shows the detailed circuit schematic of the RF front end. The LNA is based on the single-ended cascode structure with M_1,2_ (gate width = 128 μm, gate length = 60 nm) and a resistive load R_D_ (408 Ω), dissipating 520 μA. The source degeneration inductor L_s_ of 9 nH and the additional gate-to-source capacitor C_gsx_ of 0.74 pF are used for simultaneous noise and power matching. The input impedance matching is achieved only by a single off-chip inductor L_ext_ (132 nH). The G_m_ stage with M_4,5_ (gate width = 5.6 μm, gate length = 60 nm) and M_6,7_ (gate width = 16 μm, gate length = 60 nm) performs a single-to-differential conversion for interfacing between the single-ended LNA and differential mixer. It also prevents a severe degradation of the LNA performance that can be otherwise caused by the low input impedance of the passive mixer. The passive mixer with M_11,14_ (gate width = 14 μm, gate length = 60 nm) is adopted for the benefits of the low power dissipation and low 1/f noise. The differential I/Q LO signals LOI_p,m_ and LOQ_p,m_ are fed to the mixer via ac-coupling capacitors C_b_ (10 pF).

The TIA comprises an operational amplifier A_1_ and feedback components R_1_ and C_1_. The R_1_ and C_1_ are designed in a switched value structure for achieving the bandwidth tunability between 3.4 and 9.5 MHz. R_1_ is switchable among 2, 8, 9, and 10 kΩ, and C_1_ is switchable between 0 and 3.5 pF with 0.5 step. The A_1_ is a fully differential two-stage structure with its gain bandwidth product of 21.6 MHz. The total gain of the RF front end is +42.2 dB.

The IM2 calibration at the mixer is designed to minimize the IM2 distortion. It is generally known that the IM2 distortion created by FET non-linearities is manifested by the differential mismatches in FETs, dc bias voltages, passive impedances, and layout routings. Thus, the differential mismatches need be minimized to suppress the IM2 distortion at the receiver output. In this design, as proposed in [27,28], the dc gate bias voltages V_gp_ and V_gm_ for the switch FET’s M_11–14_ are controlled by employing a 6-bit R-2R digital-to-analog converter (DAC). The DAC needs to have a fine-tuning resolution for precise IM2 calibration, but a higher number of total bits will make the calibration process slow. Thus, in order to overcome the two conflicting requirements of the fine resolution and lower number of control bits, we set the full scale of the DAC to a significantly reduced value of only 50 mV, while its common-model level is tunable for a wider range between 0.5 and 0.9 V by using another 3-bit DAC. As a result, the gate bias tuning resolution is as fine as 0.78 mV. In this work, the IM2 calibration is done manually by monitoring the IM2 level with respect to the mixer gate bias voltages. In a practical mass-production stage, this IM2 calibration can be done more efficiently by using automatic test equipment (ATE).

**Figure 6:**
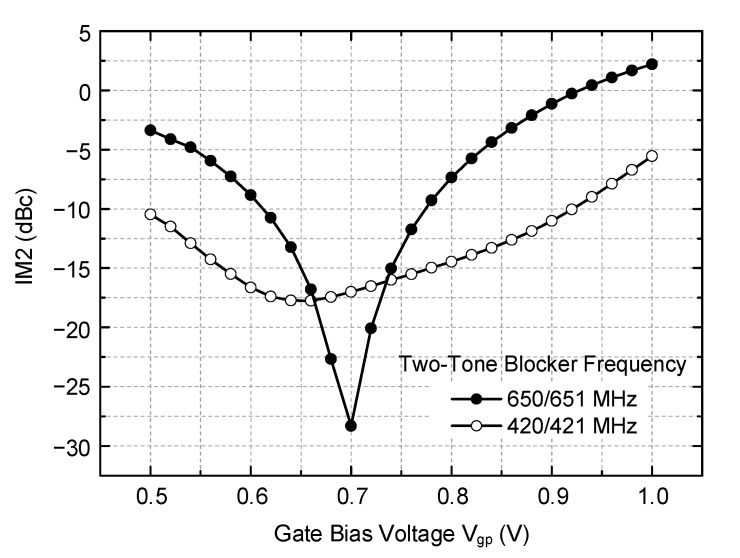
Simulation results of the IM2 calibration.

IM2 calibration is verified through circuit simulations. Figure 6 shows simulation results. The desired RF input of −30 dBm at 402 MHz is injected, and the OOB two-tone blockers of −10 dBm at (420, 421 MHz) in one simulation and (650, 651 MHz) in the other simulation are injected. With the LO frequency set to 404 MHz, the wanted IF signal appears at 2 MHz, while the IM2 distortion appears at 1 MHz. The IM2 distortion power with respect to the wanted tone power is examined with the gate bias voltage V_gp_ tuned from 0.5 to 1.0 V, while the other gate bias V_gm_ is fixed at 0.7 V. As can be seen, the IM2 distortion level is drastically reduced when the gate bias voltage V_gp_ is set to an optimal point, which is 0.65 V for 420/421 MHz blocker and 0.7 V for 650/651 MHz blocker. It clearly implies that any unwanted mismatches that are inevitable during the circuit fabrication processes can be successfully compensated by the proposed IM2 calibration circuit, thus guaranteeing a satisfactory IM2 distortion level in the receiver.

Meanwhile, it is found that the proposed IM2 calibration circuit creates an unwanted dc level change at the mixer output during the IM2 calibration. Since the signals from mixer output to the TIA, the first VGA, and the first biquad are all dc-coupled, the dc level changes at the mixer output will directly lead to a significant dc offset at the first biquad output of the complex BPF. The dc offset must be cancelled out even though the down-converted signal resides in the low-IF band and not in dc. It is because the residual dc offset created by the preceding IM2 calibration will harmfully reduce the dynamic range of the following stages. In the conventional low-IF receiver designs, for example, that reported in [12,29], the signals after the mixer output are ac-coupled. Then, the dc offset can be cancelled out by directly controlling the gate biases of the input FETs of a subsequent stage. However, in this design, that approach cannot be taken because the gate bias tuning will adversely alter the optimum IM2 calibration condition obtained at the previous mixer stage. Thus, in this work, we tune the body bias voltages of the input FETs of A_1_ to cancel out the dc offset. This approach successfully cancels the dc offset at the biquad output, while avoiding the adverse interaction with the IM2 calibration condition at the mixer.

## 4. Results and Discussions

**Figure 7:**
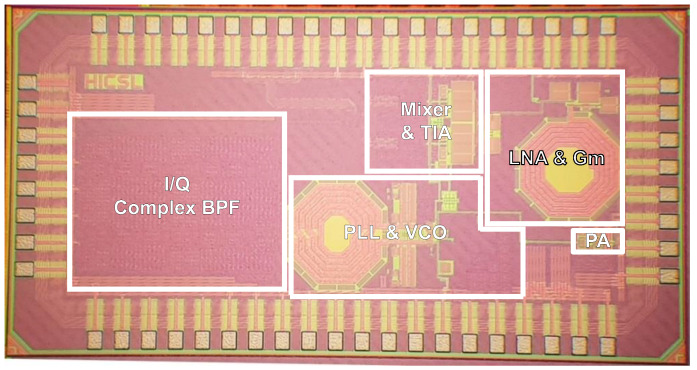
Chip micrograph.

The MedRadio receiver is fabricated in a 65 nm RF CMOS process. A micrograph of the fabricated chip is shown in Figure 7, in which the major building blocks are denotated. Note that the chip includes not only the RF receiver of Figure 3 but also an RF transmitter comprising a divide-by-4 circuit and a class-D power amplifier. However, the design details and measurement results of the RF transmitter are not discussed in this paper because they are out of the scope. Nevertheless, the total die size including the entire receiver and transmitter is 2.46 × 1.26 mm^2^. The fabricated die is mounted and directly wire-bonded on a printed circuit board for experimental measurements. A single supply voltage of 1 V is used.

**Figure 8:**
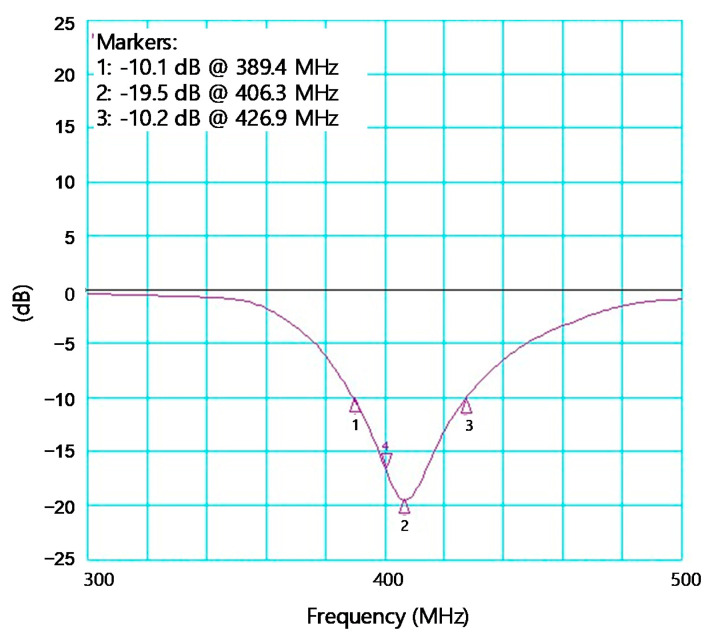
Measured S_11_ of the receiver.

Figure 8 shows the measured S_11_ of the receiver. The input impedance bandwidth having S_11_ < −10 dB is 37.5 MHz between 389.4 and 426.9 MHz. It shows that the single series off-chip inductor L_ext_ shown in Figure 5 is enough to achieve the satisfactory input bandwidth for the MedRadio applications. Note that this external L_ext_ is not changed during the subsequent whole OOB blocker measurements.

**Figure 9:**
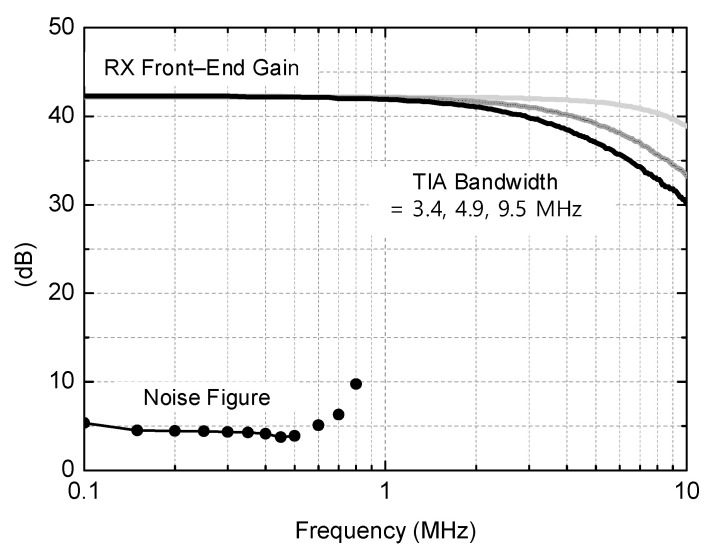
Measured gain and noise figure of the RF front end.

The measured gain and noise figure performances of the RF front end are plotted in Figure 9. The measurements were done by probing intermediate test ports at the TIA output. The gain is measured by applying the RF and LO signals by using signal generators and reading the output power level by using a spectrum analyzer (N9030B of Keysight Technologies Inc., Santa Rosa, CA, USA). The measured passband gain of the RF front end is +42.2 dB. As shown earlier in Figure 5, the on-chip feedback components R_1_ and C_1_ of the TIA are tunable. By controlling these, the TIA bandwidth is tuned between 3.4 and 9.5 MHz in eight steps. Among them, Figure 9 only displays three selected curves corresponding to the minimum 3.4 MHz, medium 4.9 MHz, and the maximum 9.5 MHz conditions for the TIA bandwidth.

For the noise figure measurement, the total output noise power *P_n,out_* with the input port terminated by 50 Ω is measured by using the spectrum analyzer. With *P_n,out_* and the receiver’s power gain G_P_ are known, the receiver noise figure is calculated by (*P_n,out_* + 174 dBm/Hz − *G_P_*). This method is convenient because it does not need a noise source or noise figure meter and also ensures sufficient accuracy because the noise floor level of the spectrum analyzer is more than 30 dB lower than *P_n,out_*. The measured noise figure at the low-IF band of 150–450 kHz is given by 3.7–4.5 dB. Assuming that a non-coherent detection for binary frequency shift keying (BFSK) signal is adopted, the minimum required SNR should be 14 dB for achieving a bit error rate (BER) of 10^−6^. In practice, the minimum BER required by a raw RF radio excluding a digital processor can be as high as ~ 10^−3^ [5], and the required SNR for this can be further lower by 3 dB, that is, only 11 dB. However, in order to take various practical errors and margins into account, we decide to use 14 dB for the minimum required SNR in the following discussions. Note that the receiver sensitivity *P_sens_* is given by

(9) $$P_{sens}=-174\left(\mathrm{dBm}/\mathrm{FHz}\right)+NF+10\log\left(B\right)+SNR_{min}$$

where *NF* is the receiver noise figure, *B* is the signal bandwidth, *SNR_min_* is the minimum required SNR. With *SNR_min_* = 14 dB, *B* = 300 kHz, and *NF* = 4.5 dB, the receiver sensitivity *P_sens_* is calculated to be −100.7 dBm or 2.06 μVrms. This sensitivity performance is comparable to [12,15] and much better than [5,13].

**Figure 10:**
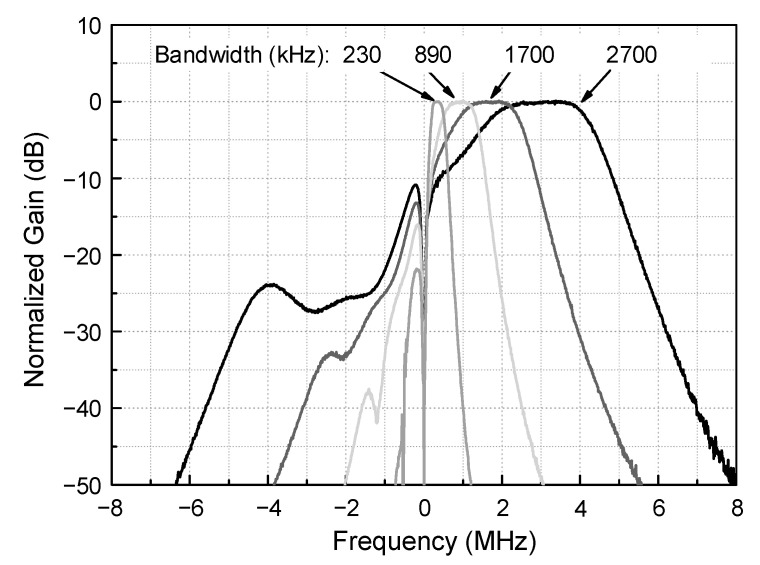
Measured frequency response of the complex BPF.

Figure 10 shows the measured frequency responses of the complex BPF. The measured curves exhibit the bandwidth tuning and image rejection performances at a medium gain condition. Although not shown here, the gain is also tunable between −22.5 and +45.4 dB in 24 steps. However, normalized gains are drawn in Figure 10 for the sake of clarity. The bandwidth is tunable between 230 and 2700 kHz in 16 steps by tuning the switched resistor and capacitor components R_1_, C_1_, R_3_, and C_3_ in Figure 4. Figure 10 only displays the four selected curves out of the total 16 curves. The image rejection ratio can be evaluated by taking the ratio of the two gain values, one at the passband’s center-frequency point and the other at its image-frequency point. The resulting image rejection ratio is 26.4–33.3 dB, which is sufficient enough considering that the minimum required SNR is 14 dB.

**Figure 11:**
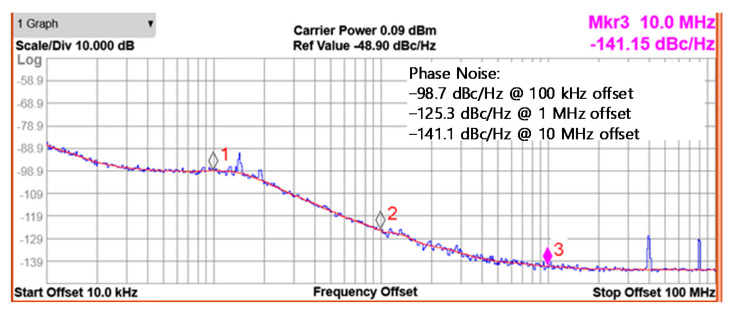
Measured phase noise of the PLL synthesizer.

Figure 11 is the measured phase noise of the PLL synthesizer at the output frequency of 400 MHz. The measured phase noise is −98.7 and −125.3 dBc/Hz at 100 kHz and 1 MHz offsets, respectively. The phase noise performance is comparable to the previously reported similar LC VCOs in [12,17], whereas it is much better than [30] for the same MedRadio applications because of the LC cross-coupled structure rather than the ring oscillator structure.

**Figure 12:**
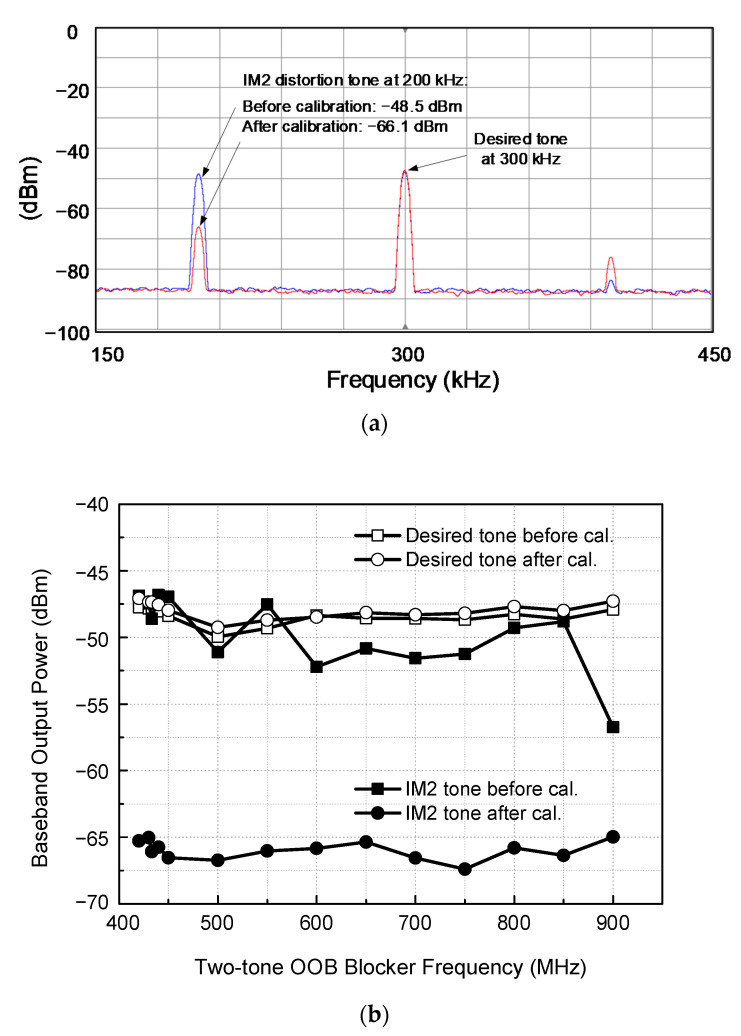
IM2 calibration performance measurements. (**a**) Spectrum, (**b**) results over the UHF band.

The IM2 calibration is verified through extensive measurements over more than five samples. All results show reasonably good agreements with acceptable variability. Figure 12a shows the measured spectrum, demonstrating how the IM2 distortion is suppressed by the proposed IM2 calibration. The desired RF single tone of −90 dBm at 403.5 MHz are fed to the receiver together with two-tone blocker of −50 dBm at 433.0 and 433.2 MHz. The receiver is set to a nominal gain condition. Then, the wanted IF and unwanted IM2 tones appear at 300 kHz and 200 kHz, respectively. Before the IM2 calibration, the desired and distortion tones are −47.8 and −48.5 dBm, respectively. After the IM2 calibration is carried out, the desired tone power does not show a noticeable change, whereas the IM2 tone power is significantly reduced from −48.5 dBm to −66.1 dBm. As a result, the signal-to-IM2-distortion ratio is improved from +0.7 dB to +18.3 dB. It implies that before the calibration, the receiver cannot satisfy the 14 dB SNR requirement, but after the calibration, it successfully satisfies the SNR requirement with a sufficient margin of 4.3 dB.

The IM2 calibration measurements are further carried out over the entire UHF band between 420 MHz and 900 MHz, and the results are plotted in Figure 12b. It is observed that the IM2 distortion is suppressed typically by 15 dB in the range of 8.3–20 dB through the IM2 calibration, while the desired tone remains almost unchanged.

**Figure 13:**
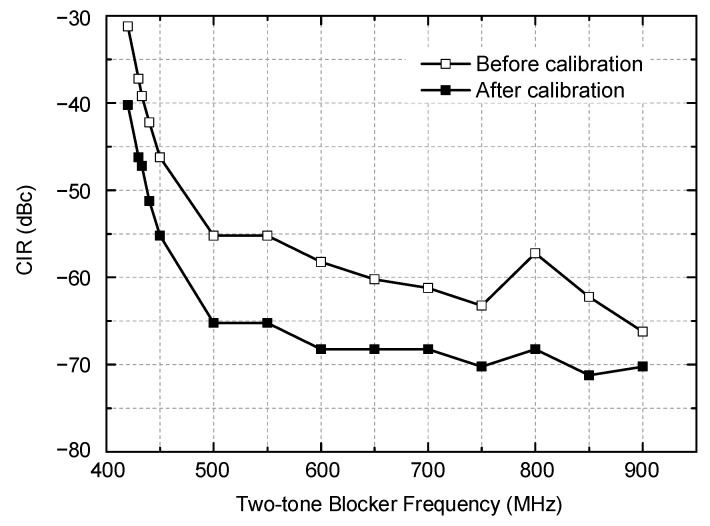
Measured maximum tolerable CIR against two-tone blocker.

The interference tolerance performances of the receiver against the OOB two-tone blocker are evaluated through the carrier-to-interference ratio (CIR) measurements and plotted in Figure 13. The maximum tolerable CIR is measured as following. First, a −90 dBm desired signal at 403.5 MHz is fed to the receiver, and an OOB two-tone blocker with 200 kHz spacing between 420 and 900 MHz is injected together. Then, the blocker signal power is raised until the output SNR reaches 17 dB. Note that we use a 3 dB higher SNR requirement for this CIR test by considering that any more noise and distortion contributions other than this IM2 distortion can be additionally involved in practice. Then, the ratio of the signal and blocker power at this 17 dB SNR condition is defined as the maximum tolerable CIR. The measured results are plotted in Figure 13. The maximum tolerable CIR indicates the receiver’s selectivity performance against the two-tone blocker. As can be seen in Figure 13, the proposed IM2 calibration effectively improves the CIR by 4–11 dB across the UHF band. The CIR after the calibration is −40.2 dBc at 420 MHz, which is very close to the in-band, and gradually improves up to −71.2 dBc as the blocker frequency moves farther away out of the band. The measured CIR can also be translated to the maximum tolerable two-tone blocker power of −49.8–−18.8 dBm. Then, considering *P_offset_* = 6.56 dB as discussed in Section 2, we can conclude that the equivalent OFDM blocker power of −43.2–−12.2 dBm can be tolerated by this RF receiver.

**Figure 14:**
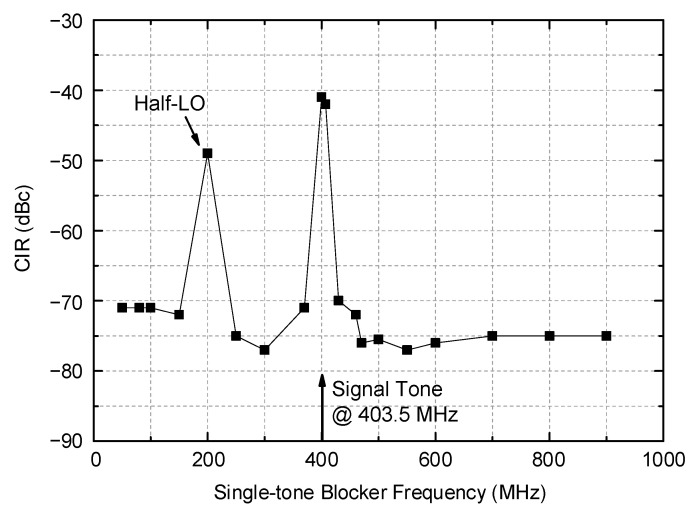
Measured maximum tolerable CIR against single-tone blocker.

Another important interference tolerance is against a single tone blocker. The measured results are plotted in Figure 14. The desired signal power is set to −98 dBm at 403.5 MHz, which is 3 dB higher than the sensitivity level. The single-tone blocker signal across the VHF and UHF band is injected together, and its power is raised up to a point such that the output SNR becomes 17 dB in the same reason described earlier. The CIR at a frequency that is only 4 MHz away from the desired tone is found to be −41 dBc, which corresponds to the maximum tolerable single-tone blocker power, is −57 dBm. However, in general, the maximum tolerable CIR shows much better performance as low as −70–−77 dBc for the rest of the band between 50–370 MHz and 430–900 MHz. One exception is observed at a half LO frequency near 200 MHz, where the CIR is observed to be −49 dBc. Although the MedRadio standard [1] does not clearly state this, such a limited set of exceptions would be generally accepted for the overall system operations because it can be avoided through high-level channel classification and a search process as is typically done in the Bluetooth receiver for internet-of-things (IoT) applications [28,29].

**Figure 15:**
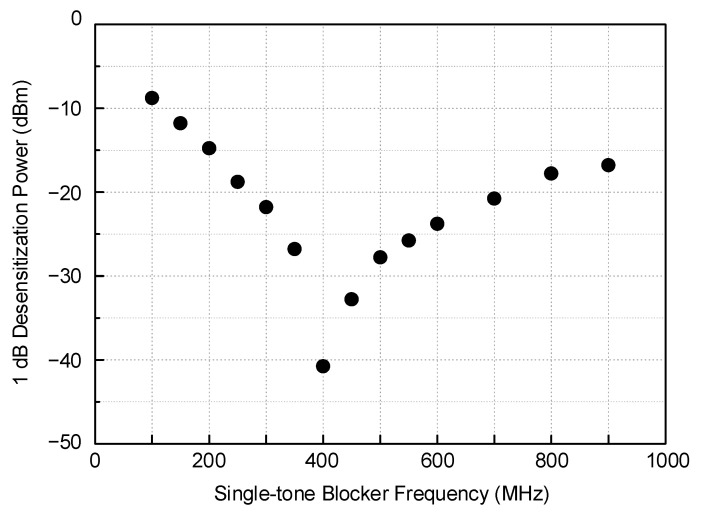
Measured single-tone 1 dB desensitization.

The 1 dB gain desensitization against the single-tone blocker is measured and shown in Figure 15. The worst point appearing at 403.5 MHz corresponds exactly to the input-referred 1 dB compression power (*IP_1dB_*) of the receiver, which is −40.8 dBm. As the blocker frequency moves away from the in-band, the 1 dB desensitization power continually grows from −26.7 dBm at 350 MHz to −9 dBm at 100 MHz for the lower band and also from −32.7 dBm at 450 MHz to −16.8 dBm at 900 MHz for the upper band.

Key performances of this work are summarized and compared with previous CMOS MedRadio receivers in Table 1. It should be noted that most of the previous works [4,5,12,14,15,16] did not address the OOB blocker tolerance performances. Only a few works reported selectivity performances. Cho et al. [18] reported an OOB CIR of −30 dBc against the VHF band interference between 30 and 70 MHz, and Ba et al. [12] demonstrated the ACR performance of 15 dB in their MedRadio receiver. Huang et al. [19] reported the CIR of −10 dB in their 915 MHz OOK receiver. Such performances should be compared to the CIR against the single tone blocker of this work. As discussed in Figure 14, this work shows much better performance, which is better than −41 dBc without an exception, or even better than −70 dBc if two exceptions are allowed at the half LO and 4 MHz away in-band frequencies. As can be seen in Table 1, this work is the first reporting the OOB CIR performances against the two-tone and single-tone blockers, which can be translated to the maximum tolerable OOB blocker power levels. Additionally, the maximum tolerable CIR against the two-tone blocker can be translated to the maximum tolerable CIR against the OFDM blocker when the *P_offset_* of Equation (8) is taken into account.

## 5. Conclusions

The OFDM signal in the UHF band for various broadcasting and communication services can severely aggravate the selectivity performance of the MedRadio RF receiver through the IM2 distortion. We have presented an analytic investigation on how the OOB OFDM signal induces the IM2 distortion and leads to the SNR degradation at the RF receiver. We also have performed theoretical analysis on how the OFDM-induced IM2 distortion can be equivalently translated to a two-tone-induced IM2 distortion. As a result, we have introduced an offset parameter *P_offset_* for compensating the difference between the multi-tone and two-tone effects. The designed MedRadio low-IF RF receiver is fabricated in a 65 nm CMOS process. Two design techniques have been described, one for the IM2 calibration through the gate bias tuning at the passive mixer’s FETs, and the other for the dc offset calibration through the body bias tuning at the subsequent TIA’s FETs. The proposed RF receivers have shown significant OOB CIR improvements, and the measured maximum tolerable CIR performances are between −40.2 and −71.2 dBc for the two-tone blocker and between −70 and −77 dBc for the single-tone blocker. To the author’s best knowledge, this work is the first to present the analytical and experimental investigations on the OFDM-induced OOB selectivity performances in the MedRadio RF receiver for the biomedical and biosensor applications. The results of this work will be essential to enhance the selectivity performance of the MedRadio RF receiver and guarantee reliable and robust wireless communication services in implanted and wearable biomedical devices.

## Figures and Tables

**Table 1:** Performance summary and comparison.

	This Work	[4]	[18]	[16]	[15]	[14]
Receiver Architecture	Low IF with complex BPF	Low IF with complex BPF	Low/Zero IF with baseband filter	Front end only	Front end only	Front end only
Gain (dB)	19.7–87.6 ^1^	32	36	31	25.7	28.7
Noise figure (dB)	4.5	5.9	-	5.2	10.2	5.5
Sensitivity (dBm)	−100.7	−85	−75	-	−97	-
IIP3 (dBm)	−24	-	-	−19.5	−17	−25
OOB CIR ^2^ by two-tone blocker (dBc)	−40.2–−71.2	-	-	-	-	-
OOB CIR ^3^ by single-tone blocker (dBc)	−70–−77	-	−30 ^4^	-	-	-
Power consumption (mW)	6.78	22	8.5	0.37	1.3	0.49
CMOS process (nm)	65	180	180	180	180	180

^1^ RF front end gain is +42.2 dB. ^2^ OOB two-tone blocker frequency at 420–900 MHz. ^3^ OOB single-tone blocker frequency at 50–370 MHz and 430–900 MHz with two exceptions at the half LO frequency and 4 MHz away in-band frequency. ^4^ OOB single-tone blocker frequency at 30–70 MHz.
